# Towards the Democratisation of Care? Insights From Co‐Governance in Local Welfare in Spain and Italy

**DOI:** 10.1111/1468-4446.70108

**Published:** 2026-03-17

**Authors:** Francesca Donati, María Jesús Rodríguez‐García

**Affiliations:** ^1^ Department of Sustainable Development and Ecological Transition University of Eastern Piedmont “Amedeo Avogadro” Vercelli Italy; ^2^ Center for Sociology and Urban Policies Pablo de Olavide University Sevilla Spain

## Abstract

The organisation and distribution of care responsibilities represent a central issue in contemporary welfare debates. Although welfare systems have progressively sought to socialise care related risks tackling distribution's inequality, the organisation of care services received less attention. The organisation of care should be democratic which requires inclusive governance that engages all stakeholders. In decentralised systems such as those in Italy and Spain, the local level plays a pivotal institutional role in shaping care policies, influenced by both vertical and horizontal subsidiarity. This article examines to what extent local governance contributes to the democratisation of care and what are the factors that shape local differences through a comparative analysis of four cities—two in Emilia‐Romagna (Italy) and two in Andalusia (Spain)—and across two policy areas: Early Childhood Education and Care (ECEC) and Long‐Term Care (LTC). Drawing content analysis of 79 policy documents and 25 interviews, we investigate the degree of care democratisation from a multilevel territorial perspective. Our findings reveal significant differences in governance between ECEC and LTC, largely attributable to supramunicipal frameworks and to the multilevel distribution of responsibilities. Regional traditions and path dependency further shape care arrangements. Nonetheless, the local dimension, particularly the political will, resources availability, and openness to participatory processes, emerge a relevant factors. We argue that national and regional institutions should reinforce municipal capacity to promote co‐governance. Moreover, enhancing the inclusion of less‐structured and marginalised actors across all levels is essential to advancing a more democratic and therefore equitable care system.

## 1 Theoretical Framework

### 1.1 Democratisation of Care

According to the literature (e.g., Tronto 2013, 2015; Kittay 2015; Orozco 2014; Federici 2020), care is a central aspect of our lives and it is essential for our survival. The provision and organization of care are fundamental to the reproduction of society and care constitutes the foundation for the sustainability of life (Orozco 2014). Despite its vital role in the functioning of society the history of care is marked by a privatisation through processes of invisibilisation, gendering, and domestication. The marginalisation of care is part of a wider centre‐periphery division, the performative and autonomous individual belongs to the center while the vulnerables and those who care for them to the periphery (Brugère 2023). Drawing on the literature (Federici 2020; Carrasco et al. 2011) we argue that the peripherialisation and marginalisation are the result of political and social choices, they are not natural or inevitable, and the organization of care can therefore be transformed. Through the primacy of the supposed impartial, rational and universal moral, the moral sentiments were confined to the private sphere (Brugère 2023). The implication was the invisibilisation of care that was confined to the private‐domestic social domain (Thomas 1993). It was framed as a duty of women characterised by love, emotion and gratuitousness (Federici 2020; McDowell 1999), which lead to an unequal distribution of care labor and responsibilities. What is relegated to the private sphere is not actively addressed by the political system, whereas what belongs to the public domain is a matter of institutional concern and action. Privatization excluded care, and the debate around care responsibilities and care needs, from the public sphere (Tronto 2013, 2015). Indeed, it limited the potential to recognise dependency and vulnerability, both of which are related to the private moral sentiments, as a central organising principle in the collective sharing of care responsibilities. This constrained the development of a fairer care system. It is acknowledged that the emergence of new social risks (Taylor‐Gooby 2004, ), particularly population aging and the entrance of women into the labor market, has produced what is referred to as a *care deficit,* compelling public institutions to socialise care risks through an active and more systematic strategy. The shift from family‐based care to private care was neither abrupt nor immediate. Public institutions gradually moved from a residual role to a more structured and systematic one, while private providers that pre‐dated the socialisation of risk continued to operate. At the same time, families often remained primarily responsible for care or continued to play a central role within broader care strategies. (Saraceno and Keck 2010). Care was partially deprivatised becoming a matter of public concern. However, while socializing care risks represents a step toward a fairer system it is only the first step. The building of a fairer care system and society requires the democratisation of care debate. In fact, the socialisation of care risks alone does not ensure the inclusion of marginalised and less heard voices in the organisation of care systems (i.e., policy/decision making). This inclusion is related the *democratisation of care* and its relevance is related to a better (and more democratic) understanding of care needs. Tronto (2013, 33) defines as a “caring democracy” a system which “requires a commitment to genuine equality of voice, and of reducing power differentials as much as possible, in order to create the conditions for a meaningful democratic discussion of the nature of responsibility in society”. The inclusion of non‐institutional voices is a try to go beyond the listening of those voices that usually regulate behaviors and frame care in all its aspects (Brugère 2023) in order to build a more democratic and fairer care system.

### 1.2 Local Governance

The local dimension of welfare within a multi‐level system gained importance with the crisis of welfare that began in the late ’70s and continued through the ‘80s. This dimension is particularly relevant in decentralized countries where welfare responsibilities are allocated to regional and local levels. Moreover, the welfare *state* crisis contributed also to the establishment of the institutional welfare mix at the local level (Ascoli and Ranci 2003). It is within this context that the public‐private governance of welfare services at the local scale became a central topic in the debate.

In this manuscript we engage with the concept of governance in line with the stream of “governing without government” (Fukuyama 2016). Our focus is on the network governance as a form of democratic participation, specifically, the involvement of non‐public actors in decision‐making processes. As Piattoni (2015) argues governance refers to the active participation of non‐governmental actors in influencing and participating to authoritative decisions rather than merely implementing or evaluating them. This shift contributes to a reconfiguration of the traditional boundary between state and society.

From this perspective, the horizontality becomes part of a strategy aimed to enhance the democratisation of welfare services (De Leonardis 1996; Navarro and Rodriguez‐Garcia 2009) and to improve them by enabling the provision services tailored to individual and territorial specific needs. Actors such as the third sector, the market, and civil society bring diverse expertise and resources, and less hierarchical collaborations can help mobilise their hidden or lost value and voices (Borzaga et al. 2024; Oosterlynck and Cools 2019; Boccacin 2009).

Nevertheless, establishing and sustaining these horizontal relationships and synergies in policymaking often proved difficult depending on the institutional capacity to foster collaborative processes (Boccacin 2009; Fazzi 2023). Public agencies can either hinder or facilitate the participation of private actors (Polizzi 2019). Therefore, co‐design and governance strategies must be guided and structured; otherwise, they risk becoming another and perhaps even more confusing way of managing policy and producing services. The role of public authority and public‐private relationships can vary depending on their specific characteristics, giving rise to different modes of governance and enabling the development of diverse different care governance systems, each with their own functioning and degree of democratisation of the policy‐making.

Against this backdrop, DiGaetano and Strom (2003) offer an analytical framework for the analysis of the local care governance that aligns with our research object. Specifically, our aim is to assess whether the local policy making in care services contributes to care democratisation by enlarging care debate and service design to non‐institutional voices. In doing so, we acknowledge the significance of the local dimension and recognise that the localisation of welfare responsibilities generates variation in local governance which may result in different degree of democratisation in care debate. DiGateano and Strom identify five types of urban governance: clientelist, corporatist, managerial, pluralist, and populist modes, each characterised by distinct **relationships between public and private actors and types of key decision‐makers**.^1^ Those elements are related to the horizontality between public and private actors and to the variety of non‐public actors included into the policy making. Both elements are related to the presence and the power of the *voices* which is central to the democratisation of care.

Their model is built on the intersection of three key elements: political actors, political culture, and structural context. These multi‐level elements interacts giving rise to different type of governance. This approach allows us to situate local governance within a broader structural and institutional framework, adopting a multi‐level perspective that includes supramunicipal levels which are relevant for the understanding of local dynamics.

Importantly, their framework accounts for territorial diversity; while contextual dimensions might be shared across regions, local political actors can interact with them in different ways, leading to varied outcomes at the local level and making it a good lens for the analysis of the multi‐level local governance.

### 1.3 Local Care Governance – The Analytical Framework

To analyze the care democratisation of the local level though the lens of local care governance we focus on the *voices* that are included in the process of policy making of care services and their *relationships*. The aim is to analyze to what extent local governments democratise care and what are the elements that foster the inclusion of different voices contributing to the democratisation of care. As explained above the democratisation of care implies the inclusion of less heard voices which also enhance citizens' capacity to make political claims regarding their care needs (Tronto 2013; Brugère 2023; Engster 2005). The participation of local actors to the policy making is important because of their role as social antennas, and thus in their ability to bring forward diverse needs. The local private actors, going beyond the public idea of care needs, can bring citizens voices to the public arena. However, their role partially depends on the public actors (De Leonardis 1996; Fazzi 2023; Polizzi 2019). In this frame DiGaetano and Strom (2003) framework offer a lens for the analysis of the openness (the degree of variety of voices included into the process) and the horizontality (the power of the voices to be heard and to influence policy making) of the policy making process. Different types of governance are related to different degree of openness and horizontality. Indeed, by observing the actors (the key decision makers) and their relationships we analyze the degree of care democratisation at the local level from a multi‐level perspective.

## 2 Material and Methods

### 2.1 Research Question

The aim of this study is to deepen our understanding of care democratisation with a particular focus on the role of the local level. To this end, we concentrate on local policy‐making within care sectors by posing the following question: *To what extent local care governance democratise care?* We analyze the degree of democratisation focusing on the variety of actors included into the policy making and the horizontality of public‐private relationships. Our central hypothesis is that the factors that foster or hinder the inclusion of different voices, generating variation among local modes of care governance, include the supramunicipal institutional environment, the political actors, and the political culture (DiGaetano and Strom 2003).

### 2.2 Methods

As stated in the introduction, we understand the local dimension as part of a multi‐level institutional system; that is, national and regional levels are relevant dimensions for understanding what occurs at the local level. Given our aim to analyze the extent to which local care governance democratises care, it is necessary to observe and analyze the role of each institutional level in shaping local care governance.

To investigate the influence of the national and regional level on local care governance, we performed a content analysis of legislative documents from both the national and the regional levels using the policy mapping approach (Lasswell 1968; Krippendorff 2018). This decision was based on twoconsiderations. First, drawing from the operationalisation of the definition of governance adopted in this paper (DiGaetano and Strom 2003; Piattoni 2015; Fukuyama 2016) the information relevant to the public‐private relationships (key decision makers and governing relationships) was available in official documents. In addition, legislative documents are the most accessible to politicians and policy makers (Kriger 2021) and offer a comprehensive basis for observing policy issues (Chambers and Bonk 2013) from a comparative perspective (Lasswell 1968).

Second, given the multi‐level organization of welfare policies, policy mapping enabled the construction of a multilevel database useful for distinguishing the role of the different institutional levels) in fostering a specific modes of governance. Following the steps identified by Bowen et al. (2022) we selected laws, decrees, and resolutions in law as of December 31, 2022, from the national and regional institutional levels. Specifically, we focused on documents related to children aged 0–3 and dependent older people. With respect to childcare, we included documents regulating both in‐kind and cash for care services, excluding social policies targeting children for reasons other than age. For dependent older people, we selected documents covering both in‐kind services such as home and residential care and cash transfer. We also defined the analytical categories to capture the key decision makers and the horizontality of the public‐private relationship established by law at the national and regional levels. With this in mind we firstly identified the mention to joint policy‐making process identifying them with the codes *co‐planning* and *co‐design.* Then we analyzed the *types of actors* allowed to participate in those process (openness) and finally the *policy tools* regulating the private‐public relationship in the policy‐making process (horizontality).

To analyze the role of the local dimension in care governance, we complemented this with semi‐structured interviews (Silverman and Marvasti 2008). This method allowed us to engage with various actors within the welfare network and to approach the interrelationships among stakeholders involved in the local system (Andreotti et al. 2012) with the aim of describing the specific urban care governance arrangements. The main advantage of semi‐structured interviews lies in their flexibility which facilitates adaptation to different informants and enhance interviewee comfort (Corbetta 1999). We asked to informants how was organised and managed the planning and design of the services. We specifically asked to each of them about the participation and the capacity and freedom to influence policy‐making and co‐designing the services of national, regional and local institutional actors, volunteer and professionalized association of the territory, civil society, and any non‐public actors. We also asked if specific instruments for joint policy making were established or used at the local level. The participation of those actors in the policy‐making process informed us about the **openness** of the care governance while their role and the instruments about the **horizontality** of those process.

In both countries, we applied the expert‐quota sampling method (Conti and Marella 2012) to ensure that the information collected was representative of the key traditional actors in the welfare mix – public actor, the market and social enterprises, and the voluntary association (Jenson 2015). We selected the institutional responsible and institutional civil servants for the two policy fields, enterprises (cooperative or market enterprises) working in the territory and volunteer associations working in the field of care with our groups of interest. This triangulation allowed to contrast and confirm the information of the actors and to ensure the saturation of the field. To confirm the reliability of the data collected through the interviews we also consulted secondary data such as local reports, official web pages and journal articles.

The analysis of the national and regional levels enabled the reconstruction of the institutional environment in which each local care governance take place with respect to supramunicipal inclusion of non‐public actors into the policy‐making and the policy tools that regulate private‐public relationships. The semi‐structured interviews, in turn, provide insights into local private‐public relationships in policy‐making allowing the analysis of how local specificities interact with the supramunicipal environment. We focused on similarities and differences between what is established at national and regional levels and what happens at the local level and create distinct urban modes of governance in care sector.

### 2.3 Case Selection

First, we selected two distinct policy fields of the same functional area (Weible 2010) care: childcare and older people. These domains represent the main challenge in care‐related welfare services. Moreover, we aim to investigate whether different degrees of care democratisation can coexist at the local level, that is whether different modes of governance emerge at the local level depending on the specific policy fields.

Second, we chose two countries: Italy and Spain. Both are historically familistic by default (Saraceno 2016), i.e., the role of public actors in the socialization of care has traditionally been limited, with care responsibilities managed primarily within the domestic sphere and with private resources. The role of the public actor was and to some extent still is limited to those cases where the families cannot take care of their dependent family members (e.g., childcare for working parents, home and residential care for those without kin network or economic resources). Additionally, both countries are regionally framed (Kazepov 2010), meaning that regional level plays a significant role in the planning and organization of welfare services. In Italy childcare and older dependent people services fall under the social services sector which were decentralised to the local level through the *Bassanini* Law No. 59 of 1997 and the 328/2000. In Spain, responsibilities for these services lies with the Autonomous Communities. Childcare services have been under regional jurisdiction since the LOGSE 1990 although in accordance with Law 7/1985 (*Bases de Régimen Local*) and Law 5/2010 (*Autonomía Local de Andalucía*) local government can request to deliver those services. Older dependent people care services are under the social services ministry are regulated by Article 92 of the Organic Law 2/2007 of 19 March and Article 9.1 of Law 5/2010 on Local Autonomy for Andalusia municipalities (over 20.000 inhabitants) which assign responsibility for the delivery of social services to municipalities. In both countries a specific system was built, in part due to historical familism and subsidiarisation.

Nowadays, despite the development of a welfare system in Italy, childcare is underdeveloped in comparison to its highly developed state in Spain. With respect to elderly care, the systems in both countries are underfinanced and lack coordination between institutional levels (Arlotti and Aguilar‐Hendrickson 2018). Indeed, the family still plays a central role in the welfare organisation of these countries. Additionally, beyond public intervention, faith‐based (Catholic) institutions have played a significant role in social services (Itçaina 2015; Tirapu 2001). According to the literature, they also prevented the development of a secular third sector and a public welfare system until the 1970s and 1980s (Naldini and Leon, 2004; Moreno and Sarasa 1992). Currently, the Church provides services within the welfare system and receives public funding, but it is no longer hegemonic, and its work align with the now developed third sector's labor in both countries (Itçaina 2015), with specific characteristics depending on the region.

Third, we selected one region in each country based on their tradition of participation and governance of welfare services: Emilia‐Romagna and Andalusia. Several studies have highlighted Emilia‐Romagna's strong participatory culture and third sector actors fabric rooted in historical traditions (Cartocci and Vanelli 2015; Cartocci 2007). Since the 60s, the Italian Communist Party (PCI) promoted local participation as a governance strategy based on localism (de Maria 2022; Bonora 1999). In this context, the PCI either founded or supported numerous associations to foster public engagement and citizen involvement (Navarro‐Yanez 1999, 168). The relationship between parties and associations is so common in Italy that authors (Ranci 1995) emphasise that the Italian third sector was not an independent actor. Nevertheless, during the 80s, the partisan participation evolved into a less political and more voluntary‐oriented participation, giving rise to a strong and more autonomous third sector fabric (Bonora 1999). Fastened by the welfare crisis and thanks to the professionalization of these they were integrated into the regional welfare system firstly under an accommodation agreement (Ascoli and Ranci 2003) and secondly under a more horizontal relationship as both providers and producers (Fargion and Gualmini 2014). In contrast, Andalusia's participatory tradition, aside from the localised neighborhood associations struggle (Becerra 2012), has been more informal and often linked to religious organisation (Prados 2009; Montero‐Gibert et al. 2006). Furthermore, the development and expansion of the welfare system in Andalucía, as in the rest of Spain, was primarily driven by the role of the public sector as a direct provider during the system's establishment (Sarasa and Obrador 2002). Furthermore, empirical studies of Spanish social services revealed a positive correlation between regional wealth and the presence of services provided by third‐sector organisations, whereas in regions such as Andalusia, externalisation was a weaker strategy (Sarasa 1995; Aguiar and Pérez 1995). Those elements limited the development and institutionalisation of a more horizontal relationship with third sector enterprises. In fact, in Andalusia public‐private relationships tend to follow a managerialist logic (Capote‐Lama et al. 2024; Cejudo‐Garcia et al., 2022), with the externalization following almost exclusively economic logic rather than the benefit of the participation of non‐public actors.

Finally, we selected four cities, two in Andalusia and two in Emilia‐Romagna. Given our understanding of the local dimension, the choice methodologically the most adequate. By comparing two cities of the same region, we are able to highlight both regional influences and territorial specificities in shaping of urban governance. We selected Bologna and Ravenna and Cádiz and Huelva based on to their levels of social spending (Wildavsky, 1964) in the two policy fields because it is related to the expansion of the welfare services and to horizontal processes (Borzaga et al. 2024). Bologna and Cádiz have higher social spending, while Huelva and Ravenna show comparatively lower levels.^2^

### 2.4 Sample

Through the policy mapping method, we selected and analyzed 79 documents – 37 from Italy and 42 from Spain – of which 30 from the national level and 49 from the regional level. Regarding the interviews following to the expert‐quota sampling method we ensured at least one interview per actor category. We conducted 25 interviews 10 with public actors, 5 with social enterprises (profit and non‐profit) and 10 with volunteer associations. The interviews were carried out from January 2023 and May 2024. They lasted between 40 and 90 min and were conducted in Italian and Spanish, depending on the interviewee.

## 3 Results and Discussion

As mentioned above this study aims to contribute to the knowledge of the democratisation of care, by focusing on local care democratisation. In this section with the help of DiGaetano and Strom (2003)'s analytical framework we present and discuss how territorial characteristics and policy fields shape distinct modes of governance and contribute to a varying degrees to the democratisation of care. We focus on the *variety of key decision‐makers (openness)* and the *public‐private relationships (horizontality)*. Building on their theory, the corporatist mode of governance implies high level of horizontality but medium openness to non‐public actors. Populist is high level of openness but a medium horizontality. Pluralist is high horizontality and high openness while managerialist is low horizontality and low openness.

Analysing the policy‐making process through the documents and interviews data we observed varying degree of openness, horizontality and, as a consequence, different types of local governance as follow in Table 1.

**TABLE 1:** Local care mode of governance.

City	Policy field	Key actors (openness)	Public‐private relationships (horizontality)	Type of governance
Bologna	Childcare sector	Politicians and professionalized actors	Horizontal	Corporatist
	Older people sector	Politicians, professionalized actors and non‐professionalized actors	Horizontal hierarchical	Corporatist and populist
Ravenna	Childcare sector	Politicians and professionalized actors	Horizontal	Corporatist
	Older people sector	Politicians, professionalized actors and non‐professionalized actors	Horizontal hierarchical	Corporatist and populist
Cadiz	Childcare sector	Politicians, professionalized actors and non‐professionalized actors	Horizontal	Pluralist
	Older people sector	Politicians, professionalized actors and non‐professionalized actors	Hierarchical horizontal	Managerialist and pluralist
Huelva	Childcare sector	Politicians and professionalized actors	Hierarchical	Managerialist
	Older people sector	Politicians and professionalized actors	Hierarchical	Managerialist

*Source:* own elaboration.

Depending on the policy fields, the city and the regional/national institutional frameworks, we observed different modes of governance in terms of the openness and horizontality of governance processes. The differences in care governance between policy fields suggest that multiple types of governance coexist within the same city. In other words, some more democratizing care processes may exist alongside less democratising ones. Differences between cities emphasise the importance of the territorial dimension, while differences between regions highlight the importance of the supra‐municipal institutional framework.

### 3.1 Childcare Sector

The local democratisation of childcare sector varies across cities with greater difference observed between Spanish municipalities. In Bologna and Ravenna local policy making align with the corporatist mode of governance described by DiGaetano and Strom (2003) which means high horizontality but a limited openness. The analysis of the legislative document informed us about this type of governance and the interviewees confirmed it. According to our informants from public institutions, social enterprises and volunteer associations working in the field of childcare, the first two confirmed their participation in policy‐making and the horizontality between them. They also confirmed that the latter were not included in policy making. This means that public and private (third sector and for profit) actors have the power to negotiate their interest but such negotiation and co‐construction of services are restricted to professionalized actors.

Building on this evidence, we asked how this horizontal relationship was built and all the informants mentioned the relevance of the *coordinamento pedagogico territoriale* (CPT). The CPT is a regional instrument (Lascoumes and Le Gales 2007) established by a national decree: decree: 65/2017 and a regional law: law: 19/2016. The aim of the CPT is to innovate the services to meet the everchanging needs of the children starting from the experience of the practitioners. It forms part of what DiGaetano and Strom (2003) identify as the institutional environment and it facilitates the public‐private collaboration between institutions and the third sector providers. The limited openness (public and professionalised non‐profit and for profit actors) and the functioning of the CPT was also confirmed through regional and local reports and data.^3^ In addition, no alternative mechanisms for the inclusion of less professionalised stakeholders in the local policy making were mentioned nor found in the documents. In light of these evidence, two key points emerge regarding the CTP. First, the democratisation of the policy‐making is limited due to the reduced openness of the process (Tronto 2013; Kittay 2015; Engster 2020) then, the local care democratisation in childcare sector is medium. Second, the CPT is a regional instrument meaning local care governance and care democratisation are directly influenced by supramunicipal institutional environment.

Nonetheless, the presence of a favourable institutional environment is not sufficient for the understanding of local care governance. Indeed, the *coordinamento pedagogico territoriale* must be “filled” with local practices. Based on the interviews with both third sector and public actors and secondary data such as the reports, the CPT appears to function successfully in both cities, with co‐planning and co‐design processes of public and private actors taking place. In this regard according to the information shared by the informants and previous literature, political culture and political actors (DiGaetano and Strom 2003), play a significant role in shaping both the regional and local approaches. Specifically, the regional path‐dependency in Emilia‐Romagna concerning the localism tradition and the co‐production of welfare policies (Bonora 1999; de Maria 2022; Navarro‐Yanez 1999) which also confirms the criteria under which the cases were selected.

Following with the cases of Cadiz and Huelva building on the openness and the horizontality (DiGaetano and Strom 2003) of care governance we argue that the mode of governance in Cadiz is pluralist (high openness and high horizontality) whereas in Huelva is managerialist (low openness and low horizontality). In Cadiz, according to the informants and confirmed by reports,^4^ horizontal policy‐making processes are organized with advocacy actors such as the *Fundación municipal de la Mujer* and third sector providers for the design of work‐life balance services other than traditional childcare. While the one municipal childcare facility is managed directly by the local government. In contrast, in Huelva, the relationship between the government and private actors delivering childcare is described as hierarchical by the informants. Specifically, contract‐based and primarily aimed at reducing public expenditure. They claim that the externalisation was not a choice, they just cannot afford the direct provision of the services. They are responsible for the management of the services and according to the legislative documents they decide what type of activities are to be provided. In this sense, the local government of Cadiz contributes more significantly than Huelva to the democratisation of care in childcare sector due to its horizontal approach and the openness to a greater variety of key actors. Conversely, the managerialist mode of governance (De Leonardis 1996) in Huelva does not foster the democratisation of care (Tronto 2013, 2015; Engster 2020). According to the information we collected, the factors shaping the mode of governance and the degree of democratisation of care policy‐making in the two municipalities include the institutional environment, the political culture and actors, economic resources and the presence of local actors.

Starting with the institutional environment (DiGaetano and Strom 2003) formal competences over the childcare lie at the regional level (regional law: 17/2007). Although municipal childcare schools exist (one in Cádiz and one in Huelva secondary data^5^ e informants report), interviewees reported no collaboration between municipal and regional nursery schools (whether public or publicly financed) and scarce collaborations between local government and regional childcare services in the territory. The regional externalization of services through the voucher system (regional law: 1/2017) has led to the creation of two separated and uncoordinated systems. At the local level the supramunicipal educational organization limits the ability of local government to establish horizontal decision making processes in care organisation because competences lie at the regional level. The territoriality fosters the collaboration in terms of interchange of resources (regional local facilities ask for resources such as the use of a park or local facilities or specific materials like chairs) as testified by the informants and secondary data^6^ but the decision‐making process are stovepipe. In light of that we can underline two facts. First, in Huelva, the hierarchical and cost‐saving externalisation reflects a managerial (functionalist) mode of governance (De Leonardis 1996) resulting in a lack of debate and no democratisation of care. According to the informants, there is no evidence of added value of the involvement or collaboration of the private actor with respect to innovation and advocacy. In addition, beyond the nursery schools, according to the legislative documents, additional services could be activated by the social services ministry (regional law: 9/2016) but they were not because of budget constraints. Second, at the regional level the institutional framework promotes the public‐private collaboration and civil society participation in the design of local services (regional law: law 9/2016). Although co‐design and co‐planning are stated as a goal of the law, no specific instrument was found in the document analysis and the lack of tools was also confirmed by the interviewees. In general, we argue that the supramunicipal institutional environment plays a significant role in limiting the local democratisation of care due to the sharing of institutional responsibilities in education sector and the lack of specific instruments does not facilitates the organisation of joint policy‐making moments.

To continue, a significant element emerged during the interviews to public actors and local associations in Cadiz and partially confirmed by the literature is the political will in Cadiz for the building of a more horizontal and democratic government. This factor is related to political actors and political culture (DiGaetano and Strom 2003). Participatory consultations have been and still are a foundational element of the party governing Cadiz: Podemos.^7^ Podemos' vision of local government is closely tied to the narrative of democratisation of local policies (Andreotti et al. 2012; Kioupkiolis 2016; Franzé, 2019), which shape the horizontality of care policy‐making supporting the democratisation of care in childcare sector. According to the informants, this pluralist mode of governance includes advocacy actors in the decision‐making process. In contrast in Huelva, the PSOE^8^ (Partido Socialista Obrero Español) exhibits a weaker participatory identity (Rivero‐Rodriguez 2018) more in line with the regional tradition of participation (Prados 2009; Montero‐Gibert et al. 2006).

Finally, the last factor we identified as central for the understanding of care governance and care democratisation is the availability of local resources. On the one side we talk about economic resources. While in Cadiz they argue that is one of the municipalities with the highest social spending in Spain in Huelva they struggled with a high inherited debt.^9^ The availability of economic resources is essential for the development of horizontal co‐design processes (Borzaga et al. 2024). On the other side, we refer to human capital. According to informants and the reports from the foundation, the *Fundacion Municipal de la Mujer in Cadiz* plays a relevant role in fostering the public‐private dialogue being a strong advocacy actor of the territory. The associative fabric described by the informants (and observed during the research) in the Andalusian municipalities is weaker than the Emilia‐Romagna's one in line with the territorial tradition of the two regions (Prados 2009; Cartocci and Vanelli 2015). In this sense the presence of this specific actor is even more relevant in a territory with a historical weaker advocacy associative fabric. In addition, regional path dependence is not related only a more or less participative culture but also to the very presence of local actors. Thus, beyond the institutional environment, local political actors and political culture, local territorial resources also shape the extent to which local care governance democratise care.

### 3.2 Older People Sector

The democratisation of care in older people policy field varies by city, with greater differences observed between Spanish municipalities. Starting with Bologna and Ravenna, we argue that the mode of governance is both corporatist and populist depending on the services being the former high horizontality but limited openness and the latter high openness but limited horizontality. DiGaetano and Strom’s (2003) categories are ideal types, but in practice, governance often takes hybrid forms; for this reason, we chose not to privilege one mode over the other.

On the one side, for the local projects, the informants of the Italian municipalities from public and volunteer entities argue that needs are selected by the public authority. They build ideas and propose them to volunteer associations for the co‐design of the realization of those ideas. This information is also confirmed by volunteer associations that argue that their influence on decision‐making is scarce, they occupy a more relevant role in providing services. On the other side, for the regional framed but local managed services, according to the interviewees (from the public institutions and the third sector) and to the legislative documents about *accreditamento,* a successful inclusion of professionalized actors (profit and non‐profit) in negotiation and policy‐making is observed. Analysing the institutional documents we observed that in Italy the supramunicipal institutional environment influences the local care governance through instruments such as the *accreditamento* (regional law: 2/2003), a policy tool (Salamon 2001) that regulates the private‐public relationship; the *Piani di Zona* (national law: 328/2000) a national instrument (Lascoumes and Le Gales 2007) for designing local socio‐health policies, and other national 328/2000, 234/2021 and 117/2017 and regional laws: 5/1994, 2/2003, 2/2014 and statue which promote negotiation between public and private actors in service design and planning.

Through the accreditamento private (profit and non‐profit) actors enter into the public welfare system ensuring the minimum standard for the delivery of services required by the public system. According to the interviews, the legislative document and to the literature (Guarna 2023) the instrument of the *accreditamento* which is designed for professionalised actors, reduces the hierarchical gap between the public and the private allowing actors to propose and experiment new types of services and to contribute with their own vision of what care should be and how care services should be delivered. Specifically, in the case of Bologna and Ravenna, both technicians, private sector actors and secondary data confirm the contamination and experimentation^10^ stated by the regional decree (Dgr 514/2009).

Nevertheless, its contribution to democratisation of care is limited because the very functioning of *the accreditamento* excludes non‐professionalized actors. Conversely, the *Piani di zona,* according to the document analysis, were designed to include less professionalized and less structured actors (law: 328/2000). However, as stated above, according to our informants from both sides (volunteer associations and technicians), this inclusion does not occur. As a result, the *Piani di Zona* resemble a populist (DiGaetano and Strom 2003) instrument for welfare governance. Which means that participation in co‐planning processes tends to be symbolic: indeed, as mentioned, institutions approach local actors to implement pre‐defined ideas, rather than engaging them in collaborative design. The democratisation of care is constrained by the fact that policy‐making is primarily led by public institutions, the horizontality is limited. The technicians and the volunteers we interviewed, and the literature (Borzaga et al. 2024; Cataldi and Gargiulo 2010) about the third sector reform and the *piani di zona,* argue that the horizontality require a level of institutional capacity that non‐professionalised actors often lack. Interviewees from the volunteer associations argue that the bureaucratic demands imposed by public authority exceed their capabilities. At the same time the public actors report difficulties in assuming responsibility for such partnerships, which sometimes makes collaboration burdensome. The third sector reform has increased the risk of excluding non‐professionalised actors (Polizzi 2019) from governance processes in the care sector, thereby complicating the effort to democratise care policies. While the supramunicipal institutional environment promotes horizontal care governance through the *Piani di zona* and the third sector reform, it simultaneously creates barriers for local institutions and actors engaged in care sector.

To continue, another element that explains the type of governance in terms of openness and horizontality is related to political actors and culture. In fact, previous studies indicate that the *Piani di Zona* have often been only partially successful (Borzaga et al. 2024; Fazzi 2023). In Bologna and Ravenna, informants highlights how strong traditions of regional and local public‐private collaboration, also confirmed by the literature (de Maria 2022; Bonora 1999), helped to mitigate some of these challenges, as for childcare sector. Thus political actors and culture are central to understating care governance, and care democratisation, as they interact with the institutional environment. Finally, according to the informants and to the documents of the *Piani di Zona*, the absence of significant differences between Bologna and Ravenna suggests that the integration of co‐*programmazione* and co‐*progettazione* as principle in Bologna's statute remains on a rhetorical dimension and care democratisation is not benefited by it compared to Ravenna.

Following with the Andalusian municipalities in Huelva, analysing the data from the interviews (public institutions, volunteer associations and private actors) we observed a hierarchical relationship between the private and the public actors. The horizontality and the openness are limited to the externalisation of the provision, care governance aligns with the managerialist mode. In Cádiz, when we asked to the informants (volunteer associations, profit and non‐profit actors, and public technicians) about joint policy‐making for care services they argue that, both formal and informal dialogues were held with third sector and civil society actors aimed at fostering horizontal policy‐making. However, the relationship with the market actor responsible for services provision, according to both public technicians and the market actor, remained hierarchical with no horizontal processes in place. Therefore, depending on the actor involved, Cadiz exhibits either a pluralist or a manageralist mode of care governance each marked by different levels of horizontality and openness of the processes. While the pluralist mode positively contributes to the democratisation of care, the manageralist does not, private actors are rather arms than partners of the public actors (Brenner, 2004). The analysis of the results points out that institutional environment plays a relevant role. Starting from the document analysis, the supramunicipal institutional environment establishes co‐governance as a guiding principle at both the national and the regional level (regional law 9/2016). However, there are no supramunicipal instruments for the institutionalization of the sharing of policy‐making in legislations. This absence gives to the municipalities the autonomy to organize the governance of care services as they prefer.

In Huelva and in Cadiz informants report that care services are outsourced through contracting out and no co‐governing process are in place. All the voices except those of public institutional are excluded. According to the literature (Guarna 2023) and confirmed by the informants contracting out instrument is based on a hierarchical relationship where public authority externalises the provision of a very specific service while the management and the policy making of the services is an exclusive public responsibility. In addition, we did not find supramunicipal instrument that could soften the hierarchy of contracting out, like for the Italian childcare sector analysing documents and interviews.

Political actors and political culture also constitute key elements in shaping care governance. In the Gatidan municipality, in addition to the policy tool (contracting‐out), another reason is cited to explain the hierarchical nature of the public‐private relationship. Specifically, public informants report the distrust of **political actors** toward the private actor in charge of local services – a French multinational corporation – believing that they it is driven only by profit. In addition, the externalisation to the French corporation is a further element highlighting the less developed history of the third sector in Andalusia in the sector of social services (Sarasa 1995; Aguiar and Pérez 1995). However, more horizontal and inclusive decision‐making processes were implemented in drafting the *plan de salud* and *plan de mayores* as both informants and public reports^11^ confirm. Both civil society associations and institutional informants confirm that a range of actors including structured volunteer organizations and informal groups were actively involved in co‐planning and co‐designing these initiatives. *Podemos* party has a **political culture** and tradition of employing participatory instruments and fostering participatory moments (Kioupkiolis 2016) and it demonstrates greater openness toward civil society and voluntary associations (though not toward the market sector) compared to PSOE administration in Huelva, thereby contributing to the democratisation of care through the inclusion of different voices (De Leonardis 1996; Brugère 2023).

Finally, another element that emerged from the interviews as relevant in shaping modes of care governance is the availability of financial resources. In Huelva, informants emphasised that there are insufficient resources to activate additional services and, consequently, to organise opportunities for exchange with local stakeholders. By contrast, in Cádiz, the narrative around public spending is linked to both the possibility and the willingness to activate services for and with the local community. Public authorities must commit to implementing the proposals that emerge from collaborative processes, otherwise those processes create distrust (Borzaga et al. 2024). To sum up we observed difference forms of care governance and varying contributions of municipalities to the democratisation of care in the field of the older people care based on the institutional environment, the decision making‐process of local political actors (DiGaetano and Strom 2003; Krueger and Park 2020) and also budgetary constraints, which can limit horizontal participation (Borzaga et al. 2024; Bartocci et al. 2023).

### 3.3 To What Extent Local Care Policy‐Making Democratise Care?

At the local level, we found different degree of care democratisation. We observed differences between cities and policy fields in terms of key actors (openness) and governing relationships (horizontality) as presented in the table above. The higher the openness and the horizontality, the higher the degree of democratisation of policy making. We calculated the degree of democratisation as the average between the degree of openness and horizontality. The results are shown in Table 2.

**TABLE 2:** democratisation of care.

City	Policy field	Type of governance	Openness	Horizontality	Degree of democratisation
Bologna	Childcare sector	Corporatist	Medium	High	Medium +
	Older people sector	Populist	High	Medium	Medium +
Ravenna	Childcare sector	Corporatist	Medium	High	Medium +
	Older people sector	Populist	High	Medium	Medium +
Cadiz	Childcare sector	Pluralist	High	High	High
	Older people sector	Managerialist	High	High	High
Huelva	Childcare sector	Managerialist	Low	Low	Low
	Older people sector	Managerialist	Low	Low	Low

*Source:* Own elaboration starting from di Gaetano and Strom (2003) categories.

These results suggest that local policy‐making democratise care, albeit to differing degrees, with variations between municipalities and policy fields reflecting inequalities in the extent to which their care systems are democratised.

In addition, comparing two policy fields across 4 municipalities certain patterns emerge allowing us to analyze the elements that foster and hinder policy‐making democratisation which in turn lead to different degree of democratisation of care.

In our case studies, the lowest contribution to care democratisation was observed in Huelva while the highest in Cadiz. In the latter, the co‐governance processes were highly horizontal and open in both policy fields. In contrast, Huelva exhibited more hierarchical and exclusive governance. Bologna and Ravenna occupy an intermediate position between these two extremes. In both cities the older people care field showed more democratic features than childcare field where negotiations were more exclusive, higher and lower openness, respectively. Summarising and linking this evidence, we argue that a certain hierarchy exists among the elements influencing the degree of local care democratisation.

1. The distribution of formal responsibilities
2. Supramunicipal instruments
3. Political will, political culture and economic resources
4. The presence of territorial actors.

First, the absence of formal responsibilities limits co‐governance processes for the childcare in Cádiz and Huelva regardless of other local specifics. Second, supramunicipal instruments institutionalise horizontal governance processes at the local level. While the local dimension is key to understanding the outcomes of these mechanisms, the lack of such instruments can result in a complete lack of horizontal governance, as seen in Huelva. Third, political will, political culture and economic resources are important elements. Political will can compensate for a less nstitutionalized governance framework, as in Cádiz, and enable horizontal processes. On the contrary, economic resources are indispensable: even with strong political will, a lack of funding can prevent the activation of these processes. Lastly, the presence of local actors, such as third‐sector organization (which is also related to the local and regional history of third sector), can foster the democratisation of care, as observed in Cádiz and Bologna and Ravenna, but their presence alone is not insufficient to ensure of horizontal governance. To conclude, this study also reveal that it is inaccurate to speak about the democratisation of care debate and policies as monolithic process, given the differences between the policy fields especially shaped by the sharing of formal responsibilities and supramunicipal instruments. Broadly speaking, care, still faces substantial challenges on this path toward democratisation. Bringing care into the public debate is essential to avoid treating it as a private or individual issue. Encouraging public discussion about care fosters collective responsibility and a more democratic understating of care and care needs (Tronto 2013; Brugère 2023) in other words a more democratic care system.

## 4 Conclusions and Implications

We analyzed the mode of care governance in four municipalities and two policy fields observing that different degrees of care democratisation exist and coexist. These differences between the cities (and the similarities between regions) are primarily shaped by supramunicipal factors such as the allocation of formal responsibilities and the presence of national or regional instruments for co‐design and co‐planning processes. In addition, local governance, in decentralised countries, could stem from regional traditions and path dependency. Finally, the availability of local resources, political will, and the local traditions also play a crucial role.

Based on this evidence and considering the importance of local governance for both the democratisation of care and the public–private tailoring of services we suggest that supramunicipal institutional levels should strength municipal capacities for local strategic governance. National or regional instruments should offer a general framework that is adaptable to local specificities while also facilitating the establishment of co‐governance processes in care policy fields. Furthermore, in light of the resource constraints observed, increased financial support for local policies would significantly enhance the democratisation of care by enabling more horizontal governance arrangements. In addition, the creation of territorial social enterprises should be promoted especially in those regions where the third sector fabric is weaker. Supramunicipal levels should ensure that municipalities are adequately resourced and supported empowering civil servants to promote and sustain horizontal co‐governance processes. To conclude, mechanisms for the inclusion of less structured actors should be developed across all institutional levels to reinforce the democratic potential of co‐governance in care sector. Including historically marginalised voices is essential for building a democratic and a more equitable care system especially considering the current shift from welfare state to community welfare. There cannot be a new community welfare if the voice of the community is not listened for the building of the system.

## Funding

Data collection was sponsored by the project DE‐FAMILIZATION AT THE LOCAL LEVEL. PUBLIC POLICIES AND EFFECTS ON HOUSEHOLD CARE STRATEGIES, funded by the Department of Economic Transformation, Industry, Commerce and Universities. Regional Government of Andalucía (PY20_00827).

## Consent

Informed consent was obtained from all individual participants.

## Conflicts of Interest

The authors declare no conflicts of interest.

## Data Availability

The data that support the findings of this study are available from the corresponding author upon reasonable request.

## Endnotes

^1^
The clientelistic mode is characterized by particularistic relationships and interests guided by the principle of reciprocity. The key actors are politicians and clients. The corporatist mode: public private relationships are based on exclusionary negotiations aimed at building consensus. The key actors are politicians and powerful civic leaders. The managerialist mode of governance is oriented toward on efficacy and efficiency. Public‐private relationships rely on contractual and bureaucratic tools designed to support authoritative decision making. Key actors are politicians and civil servants. The pluralistic mode: public‐private relationships are based on the mediation of competing interest with the goal of managing conflict Key actors are the politicians and the organized interests. The populistic mode: private‐public relationships are based on inclusionary negotiations aimed at mobilising popular support. Key actors are the leaders of community movements.

^2^
The Spanish municipalities were selected according to the 2021 data from the “Informacion de los presupuestos de las entidades locales” of the Ministerio de Hacienda. For the Italian selection, we used the 2021's “Interventi e servizi sociali dei comuni” data from ISTAT (Istituto Nazionale di Statistica).

^3^
Bologna's CTP: https://cptbologna.it/chi‐siamo/; Ravenna's CTP: https://comune.ravenna.it/wp‐content/uploads/2025/09/Progetto‐pedagogico‐dei‐servizi‐per‐linfanzia‐0‐6‐anni‐del‐Comune‐di‐Ravenna.pdf Regional report: https://sociale.regione.emilia‐romagna.it/nidi‐e‐scuole‐dellinfanzia/coordinamenti‐pedagogici.

^4^

https://adelantecadiz.info/proyectos/cadiz‐de‐cuidados/; https://transparencia.cadiz.es/el‐ayuntamiento‐pone‐en‐marcha‐dos‐ludotecas‐permanentes‐para‐favorecer‐la‐ conciliacion‐familiar/.

^5^

https://institucional.cadiz.es/area/Escuela‐Infantil‐Municipal‐%22SANTA‐MARÍA%22/60; https://www.eimonigoteslahispanidad.es.

^6^

https://institucional.cadiz.es/iframe/convenios.

^7^
Podemos was ruling Cadiz in 2023 when the interviews were carried out.

^8^
The PSOE was ruling in Huelva when the interviews were carried out.

^9^
327.661.500 euros of debt (indicadores financieros, patrimoniales y presupuestarios del Ayt. De Huelva a la fecha 14/10/2016). The average difference between Cadiz and Huelva for the period 2019–2023 in social spending in of 50 euros per capite: https://serviciostelematicosext.hacienda.gob.es/SGFAL/CONPREL.

^10^
An example: “CRA Aperta” https://www.ausl.bologna.it/news/archivio‐2024/presentato‐il‐progetto‐cra‐aperta‐la‐casa‐residenza‐anziani‐beata‐vergine‐al‐servizio‐della‐comunita.

^11^

https://transparencia.cadiz.es/el‐ayuntamiento‐inicia‐la‐creacion‐de‐un‐plan‐municipal‐para‐personas‐mayores/; https://www.redlocalsalud.es/buenaspracticas/documentos/material1_68.pdf.
